# A new soaking procedure for X-ray crystallographic structural determination of protein–peptide complexes

**DOI:** 10.1107/S2053230X2001122X

**Published:** 2020-09-15

**Authors:** Alice Ballone, Roxanne A. Lau, Fabian P. A. Zweipfenning, Christian Ottmann

**Affiliations:** a Laboratory of Chemical Biology, Department of Biomedical Engineering and Institute for Complex Molecular Systems, Eindhoven University of Technology, Den Dolech 2, 5612 AZ Eindhoven, The Netherlands; b Department of Chemistry, University of Duisburg-Essen, Universitätsstrasse 7, 45117 Essen, Germany

**Keywords:** co-crystallization, crystal soaking, protein–peptide complexes

## Abstract

Interactions between a protein and a peptide motif of its protein partner are prevalent in nature. Often, a protein also has multiple interaction partners. X-ray protein crystallography is commonly used to examine these interactions in terms of bond distances and angles as well as to describe hotspots within protein complexes. However, the crystallization process presents a significant bottleneck in structure determination since it often requires notably time-consuming screening procedures, which involve testing a broad range of crystallization conditions via a trial-and-error approach. This difficulty is also increased as each protein–peptide complex does not necessarily crystallize under the same conditions. Here, a new co-crystallization/peptide-soaking method is presented which circumvents the need to return to the initial lengthy crystal screening and optimization processes for each consequent new complex. The 14-3-3σ protein, which has multiple interacting partners with specific peptidic motifs, was used as a case study. It was found that co-crystals of 14-3-3σ and a low-affinity peptide from one of its partners, c-Jun, could easily be soaked with another interacting peptide to quickly and easily generate new structures at high resolution. Not only does this significantly reduce the production time, but new 14-3-3–peptide structures that were previously not accessible with the 14-3-3σ isoform, despite screening hundreds of other different conditions, were now also able to be resolved. The findings achieved in this study may be considered as a supporting and practical guide to potentially enable the acceleration of the crystallization process of any protein–peptide system.

## Abstract 2

A combined co-crystallization/crystal-soaking procedure has been devised for the efficient structural elucidation of protein–peptide complexes.

## 1. Introduction   

Ample protein–protein interactions (PPIs) that occur within the cell are formed between proteins that contain peptide-binding domains and the short peptidic motifs of their partner proteins (Cunningham *et al.*, 2020 ▸). Decoding the 3D structure of a protein–peptide complex by using state-of-the-art X-ray protein crystallography is essential to obtain snapshots of the final state of the interactions within the complex and provides adequate insights into inter-residue interactions, the stability of protein structures and the recognition mechanism of the protein–peptide system (Gromiha *et al.*, 2019 ▸), as well as determining potential ‘druggable’ hotspots in the context of drug discovery (McIntyre *et al.*, 2017 ▸). However, such interactions are numerous, thereby placing a significant burden on crystallization studies, which are normally a time-consuming process (Cunningham *et al.*, 2020 ▸). In this regard, protein crystallization is consistently the rate-limiting step in structure determination, with strategies often depending on pure luck via the screening of hundreds or thousands of conditions for the observation of crystal growth (Smyth & Martin, 2000 ▸; Gorrec, 2016 ▸; D’Arcy, 1994 ▸). In addition to this, there is not a single standard method to successfully crystallize a specific protein–peptide complex (Müller, 2017 ▸). Similarly, when a protein structure is effectively crystallized with a peptide, it is not always straightforward to reproduce the same crystal with another peptide.

We have observed this phenomenon first-hand with human 14-3-3σ, a protein which has several hundred known interacting partners in the cell (Johnson *et al.*, 2010 ▸). These partners bind to 14-3-3 via specific peptidic motifs, which can be classified into three modes: (i) RS*X*(pS/T)*X*P, (ii) R*X*(F/Y)*X*(pS)*X*P (Yaffe *et al.*, 1997 ▸) and (iii) C-terminal sequences (pS/T)*X*_1–2_-COOH (Coblitz *et al.*, 2006 ▸). So far, we have found that the apo form of 14-3-3σ is not able to crystallize by itself. However, structure determination of 14-3-3σ in complex with a chemically synthesized peptide containing one of these modes from one of its interacting partners is a process that has been performed repeatedly, with many examples being found in the PDB (Anders *et al.*, 2013 ▸; Joo *et al.*, 2015 ▸; Schumacher, Skwarczynska *et al.*, 2010 ▸; Schumacher, Mondry *et al.*, 2010 ▸; Rose *et al.*, 2012 ▸; Molzan *et al.*, 2012 ▸). These 14-3-3σ crystals were optimized to grow under in-house ‘sigma’ grid-screening conditions (Table 1 ▸), which were derived from Qiagen JCSG Core I–IV screens (Qiagen, Hilden, Germany; Schumacher, Skwarczynska *et al.*, 2010 ▸), where they were observed to grow to a suitable size and shape within five days and diffract to high resolution with high quality. Furthermore, these crystals are robust and stable, thereby making them very amenable to fragment and compound soaking (for example, by being able to withstand the high concentrations of dimethyl sulfoxide that such compounds are regularly dissolved in; Guillory *et al.*, 2020 ▸).

The challenge that we have found is that while many of the peptides from 14-3-3-interacting partners synthesized so far have been able to co-crystallize with 14-3-3σ in the optimized ‘sigma’ conditions (Bier *et al.*, 2016 ▸; Ballone, Centorrino, Wolter *et al.*, 2018*b* ▸; Stevers *et al.*, 2018 ▸), there are still a significant number of other peptides that have resisted this path. For these, structure determination has required going back to the lengthy initial screening and crystal-optimization processes, and frequently these crystals, if they do manage to grow, are not robust enough to withstand the fragment/compound-soaking process. In addition to this, there have also been many peptides for which structure determination with the 14-3-3σ isoform has not yet been possible despite carrying out hundreds of screening conditions in addition to our optimized ‘sigma’ conditions (Supplementary Table S1).

To address this problem, we present a combined co-crystallization and peptide-soaking approach to obtain crystals for any 14-3-3σ–peptide complex that may exhibit difficulties when crystallizing under our optimized ‘sigma’ conditions. This new approach (i) permits the obstacle of the identification of suitable new conditions for the crystallization of specific 14-3-3–peptide targets to be omitted, thereby significantly reducing the time and resources that are needed to produce these crystals, (ii) allows an atomic model of a 14-3-3σ protein–peptide system not explored previously to quickly be obtained and (iii) retains the quality of the crystals grown under the ‘sigma’ conditions, thereby providing an excellent starting point for screening fragment and compound libraries.

The methodology requires two main steps. The first step involves the growth of 14-3-3σ crystals in complex with a weakly binding c-Jun peptide (cJun-pS227; Supplementary Fig. S2) under the aforementioned ‘sigma’ conditions (Table 1 ▸). Structure determination of this complex reveals electron density that is not observable for most of the c-Jun peptide, with only weak electron density appearing for its phosphorylated serine site (Fig. 2*a*). The second step involves, after these 14-3-3σ–cJun-pS227 crystals are fully grown, displacement of the c-Jun peptide via addition of the desired peptide for the final structure at high concentration to the crystal drop and incubation for a period of time until full occupancy of the peptide is achieved.

To demonstrate the experimental reliability of the method, we considered this approach for peptides (Supplementary Table S1) found in the PDB where co-crystal structures were published with isoforms of 14-3-3 other than the sigma isoform, since they were not able to crystallize under the standard ‘sigma’ conditions or any of the other hundreds of screening conditions tested. Data collection, performed at Diamond Light Source, Oxford, UK or at our home source, was successful for all of the trial cases, resulting in a particularly encouraging new methodology for expanding our knowledge in the structural analysis of PPIs.

## 2. Materials and methods   

All chemically synthesized peptides were obtained from GenScript, USA (Supplementary Table S1). All peptides were acetylated at their N-termini.

C-terminally truncated 14-3-3σ (ΔC, devoid of the last 18 C-terminal residues) was expressed using *Escherichia coli* BL21(DE3) cells employing pPROEX HTb expression plasmids and purified via a nickel column. After purification, the His_6_ tag was cleaved with TEV protease and a second purification was performed by size-exclusion chromatography as described previously (Schumacher, Skwarczynska *et al.*, 2010 ▸).

The 14-3-3σ protein was mixed with cJun-pS227 in a 1:1 molar ratio in complexation buffer (20 m*M* HEPES, 2 m*M* MgCl_2_, 2 m*M* β-mercaptoethanol) to a final concentration of 12 mg ml^−1^ protein. The complex was incubated on ice for an hour for crystallization. Screening for crystallization was performed by the sitting-drop vapour-diffusion method at 277 K with in-house-made sigma grid screening (Table 1 ▸) using a Mosquito Crystal Nanolitre Protein Crystallization robot (SPT Labtech) and a final volume of 500 nl (250 nl protein–peptide mixture and 250 nl well solution).

All peptides were dissolved in complexation buffer to make a 5 m*M* stock and were injected into the drops containing the 14-3-3σ–cJun-pS227 crystals to a final concentration of 3 m*M* peptide and a final volume of 1.25 µl. These were resealed and were left to incubate for one week at 277 K before crystal harvesting. Crystals were harvested and cryocooled by direct transfer from the mother liquor to liquid nitrogen.

Diffraction data were processed using the *DIALS xia*2 package (Winter, 2010 ▸). The structures were solved by molecular replacement in *Phaser* (McCoy *et al.*, 2007 ▸) using PDB entry 3lw1 as the search model (Schumacher, Mondry *et al.*, 2010 ▸). These models were then subjected to improvement via iterative rounds of model building and refinement with isotropic *B* factors using *Coot* (Emsley *et al.*, 2010 ▸), *REFMAC*5 (Kabsch, 2010 ▸) and *Phenix* (Liebschner *et al.*, 2019 ▸). Structures were validated with *MolProbity* (Williams *et al.*, 2018 ▸). Figures were created using *CCP*4*mg* (McNicholas *et al.*, 2011 ▸).

## 3. Results   

We have found useful criteria to be using the peptide at >95% purity and performing the soaking at 277 K with an incubation time of one week. A peptide sample at a final concentration of 3 m*M* is a helpful starting point, as well as a final purified 14-3-3σ ΔC protein concentration of 12 mg ml^−1^.

The condition most suitable for crystal growth was determined to be 28%(*v*/*v*) PEG 400, 5% glycerol, 0.2 *M* CaCl, 0.1 *M* HEPES pH 7.5, 2 m*M* β-mercaptoethanol and was used for reproduction of the crystals. Well diffracting and robust crystals grew in between one and two weeks to sizes of between 0.1 and 0.3 mm.

X-ray diffraction data sets were collected at 100 K on the I03 and I24 beamlines at Diamond Light Source, Oxford, UK or using our Rigaku MicroMax-003 home source. In the first case, crystal data were measured at a wavelength of 0.9763 Å using a Dectris EIGER2 XE 16M detector. At our home source, crystal data were measured at a wavelength of 1.541 Å using a Dectris PILATUS 200K detector.

**Figure 1:**
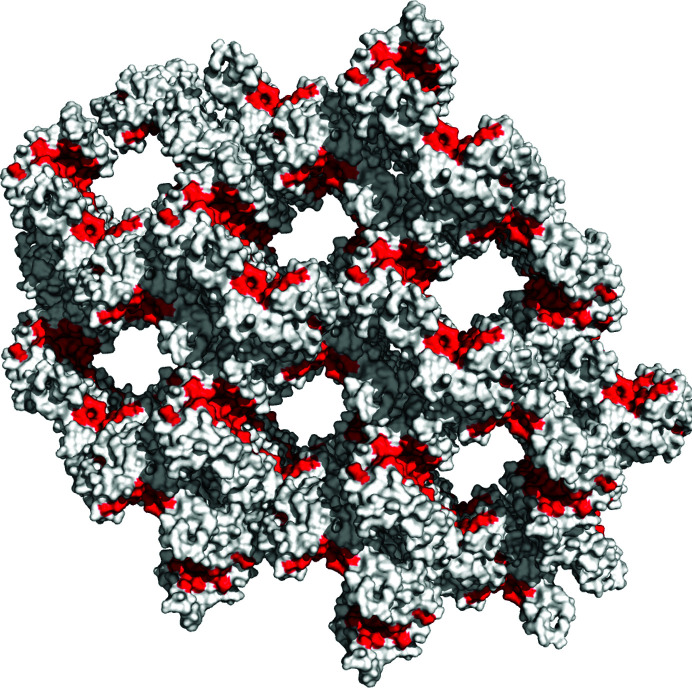
The crystal lattice of the 14-3-3σ–cJun-pS227 structure. The figure, which was created in *PyMOL* version 1.2r3pre (Schrödinger), shows the protein in white with its binding groove shown in red.

All models were determined to belong to the orthorhombic space group *C*222_1_ using *Zanuda* (Lebedev & Isupov, 2014 ▸). The crystal lattice of the 14-3-3σ–cJun-pS227 structure (Fig. 1 ▸) shows that the amphipathic binding groove of the 14-3-3 protein is easily accessible for peptide exchange via solvent-filled channels.

**Figure 2:**
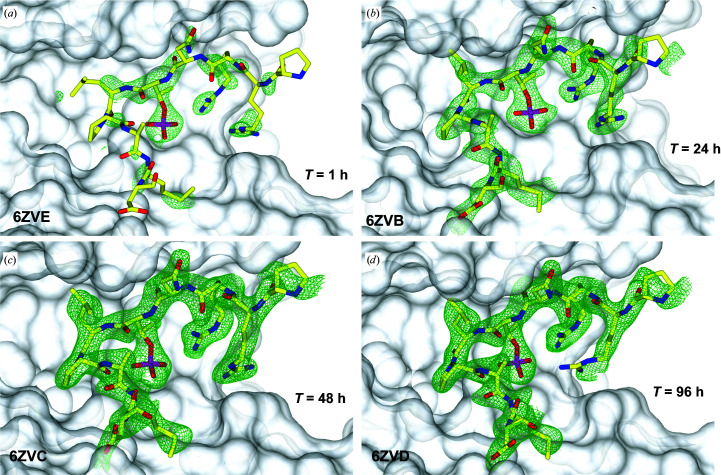
Time series of Gab2-pT391 replacing cJun-pS227 in the amphipathic groove of 14-3-3σ over (*a*) 1 h, (*b*) 24 h, (*c*) 48 h and (*d*) 96 h. Growth of electron density for the Gab2-pT391 peptide is observed over time. The peptide is represented by yellow sticks. 14-3-3 is represented by a white, solid surface. The *F*_o_ − *F*_c_ electron-density map (represented by a green mesh) around the peptide is contoured at 2.4σ. Refined occupancies for the modelled amino acids of the Gab2-pT391 peptides are plotted in Supplementary Fig. S1.

**Figure 3:**
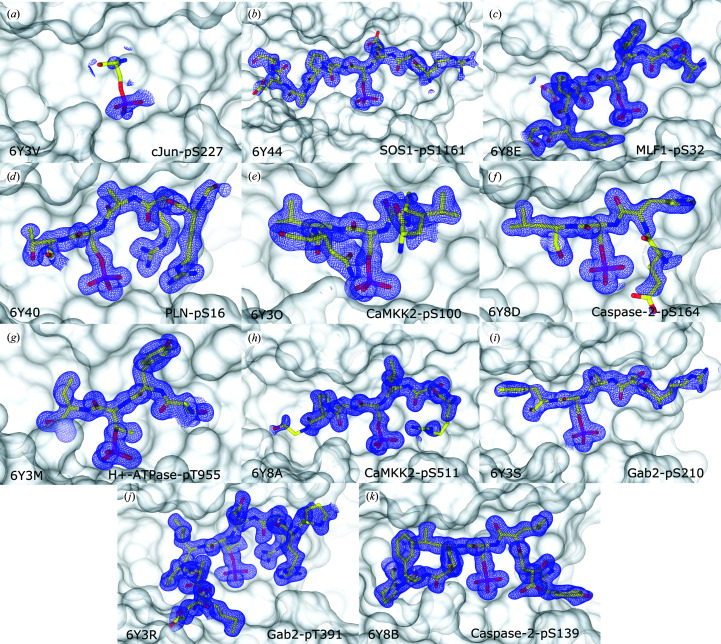
14-3-3–peptide complexes before and after soaking 14-3-3–cJun-pS227 crystals with a different peptide. (*a*) The 14-3-3–cJun-pS227 complex before soaking with another peptide. After soaking with another peptide, structures were solved for 14-3-3–peptide complexes containing the (*b*) SOS1-pS1161, (*c*) MLF1-pS32, (*d*) PLN-pS16, (*e*) CaMKK2-pS100, (*f*) caspase-2-pS164, (*g*) H^+^-ATPase-pT955, (*h*) CaMKK2-pS511, (*i*) Gab2-pS210, (*j*) Gab2-pT391 and (*k*) caspase-2-pS139 peptides. Peptides are represented by yellow sticks. 14-3-3 is represented by a white, solid surface. The 2*F*_o_ − *F*_c_ electron-density maps (represented by a blue mesh) around the peptides are contoured at 1σ.

We observed the growth in electron density for such a peptide (Gab2-pT391) over several time points, achieving full occupancy by 48 h (Figs. 2 ▸ *c* and 2 ▸ *d*). Because of the near-zero electron density observed for the c-Jun peptide (Fig. 3 ▸ *a*), any positive electron density in the amphipathic groove of 14-3-3σ observed after soaking can be confidently attributed to the new peptide.

**Figure 4:**
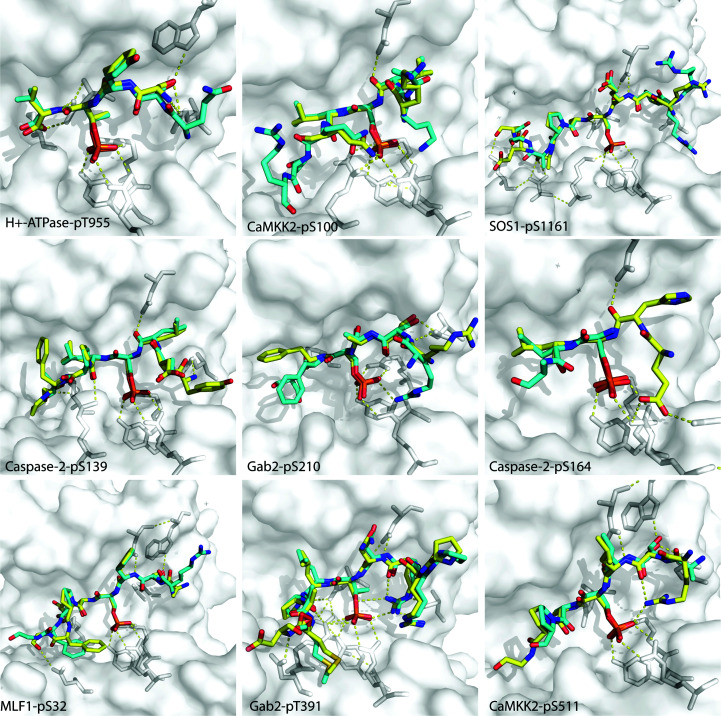
Superimposition of 14-3-3σ–peptide complexes derived from the c-Jun peptide soaking methodology with the equivalent crystal structures previously deposited in the PDB [PDB (1) and PDB (2), respectively; see Supplementary Table S1]. 14-3-3σ is represented as a white transparent surface and white cartoon. PDB (1) peptides are represented as yellow sticks and PDB (2) peptides are represented as cyan sticks. Polar contacts between the peptide and protein are represented by yellow dashed lines. This figure was created in *PyMOL* version 1.2r3pre (Schrödinger).

Structure determination with 14-3-3σ was fruitful for each of the ten new structures that we present in this study, each with good observable electron density for the peptide (Figs. 3 ▸ *a*–3 ▸ *k*). With the exception of the case of the PLN-pS16 peptide, for which structural determination in complex with other 14-3-3 isoforms had not been achieved previously using X-ray crystallography, all of the structures reported in this study are very similar to those already published in the PDB (Fig. 4 ▸; Bier *et al.*, 2016 ▸; Molzan *et al.*, 2012 ▸, 2013 ▸; Psenakova *et al.*, 2018 ▸; Ballone, Centorrino, Wolter *et al.*, 2018*a* ▸,*b* ▸), providing proof-of-principle validation.

## 4. Discussion and conclusions   

X-ray crystallography continues to remain the gold standard for protein structure determination. However, the technique does not come without limitations, and one major bottleneck lies within the crystallization process itself. This process has always relied on cumbersome trial-and-error practices. The search for conditions for protein crystal growth is typically a long and arduous process, requiring the need to screen a multitude of different buffer combinations, with many frustrations along the way. Owing to this, X-ray protein crystallography is well known to be a technique that may take from weeks to months or, in extreme cases, even years to find the right conditions to crystallize a certain protein complex.

The new strategy that we present here has allowed the determination of several new structures of 14-3-3σ which had not previously been possible, illustrating the ease and success of the method. We therefore expect that this can be applied to all further difficult-to-crystallize 14-3-3σ–peptide complexes. In addition, the method retains the robust, stable, high-resolution and well diffracting features of crystals originally grown in the sigma screen. The growth of such crystals is enormously valuable because 14-3-3 protein–protein inter­actions have been studied during the past decade as suitable targets for both inhibition or stabilization (Skwarczynska & Ottmann, 2015 ▸). Alternative methods presented previously (Sluchanko *et al.*, 2017 ▸) are not as straightforward as the methodology presented here since they require steps such as the design, expression and purification of chimeric proteins. Furthermore, different isoforms of the 14-3-3 protein are function-specific. For example, the 14-3-3σ isoform (but not other isoforms) specifically targets p53 and is associated with tumour suppression (Benzinger *et al.*, 2005 ▸), making it invaluable to determine crystal structures of the target peptide with the correct isoform (Benzinger *et al.*, 2005 ▸).

These 14-3-3σ–peptide co-crystal structures have provided the basis for early drug-discovery projects, representing structural proofs from a druggability perspective and leading to the identification of starting-point modulators for PPIs (Sijbesma *et al.*, 2017 ▸, 2019 ▸). So far, promising starting points have been achieved. An example is given by several findings which have shown that compounds such as fusicoccin A (Stevers *et al.*, 2016 ▸) or fusicoccin derivatives (Bier *et al.*, 2016 ▸) can be used to target and modulate the interface of 14-3-3–peptide complex interfaces, emphasizing the potential druggability of these systems (Ballone, Centorrino & Ottmann, 2018 ▸).

With a large number of PPIs involving 14-3-3 (Johnson *et al.*, 2010 ▸) identified as potential therapeutic targets, this approach can determine the structures of these complexes in a straightforward manner and therefore will assist greatly in the first steps of structure-based drug development, producing crystals from which structures can easily be determined and which can be used as a basis for soaking fragments/compounds (both inhibitors and stabilizers of 14-3-3 PPIs; Stevers *et al.*, 2018 ▸; Ballone *et al.*, 2018 ▸). So far, discovered small-molecule ligands of 14-3-3 proteins display a considerable degree of chemical diversity, ranging from fragments (Sijbesma *et al.*, 2017 ▸, 2019 ▸) and ‘classical’ small molecules to supramolecular ligands (Bier *et al.*, 2013 ▸, 2017 ▸; de Vink *et al.*, 2017 ▸) and natural products (Andrei, de Vink *et al.*, 2018 ▸; Kaplan *et al.*, 2020 ▸; Molzan *et al.*, 2013 ▸) to modified peptides (Glas *et al.*, 2014 ▸; Andrei, Meijer *et al.*, 2018 ▸; Milroy *et al.*, 2015 ▸; Andrei *et al.*, 2019 ▸), with most requiring specific co-crystallization conditions. Our new method may also enable a faster and more modular elucidation of these ligands in the future. This approach could also potentially be used for application to other protein–peptide complexes where (i) a low-affinity peptide that co-crystallizes with the protein can be identified and (ii) the binding site is accessible within the crystal form (Fig. 1 ▸).

In conclusion, our approach is able to overcome the crystallization bottleneck when trying to determine structures of the same protein in complex with different peptides, thus offering new possibilities for the fields of PPIs and structure-based drug design. In structural biology these findings may have important implications for targets that have not yet been structurally characterized, where the experimental identification of relevant binding sites can simplify the development and optimization of new tool drug-like molecules that are able to modulate these protein–protein complexes.

## Supplementary Material

Supplementary Tables and Figures. DOI: 10.1107/S2053230X2001122X/nj5291sup1.pdf

PDB reference: 14-3-3σ, complex with cJun-pS227, 6y3v

PDB reference: complex with CaMKK2-pS100, 6y3o

PDB reference: complex with CaMKK2-pS511, 6y8a

PDB reference: complex with caspase-2-pS139, 6y8b

PDB reference: complex with caspase-2-pS164, 6y8d

PDB reference: complex with Gab2-pS210, 6y3s

PDB reference: complex with Gab2-pT391, 6y3r

PDB reference: complex with H^+^-ATPase-pT955, 6y3m

PDB reference: complex with MLF1-pS32, 6y3e

PDB reference: complex with SOS1-pS1161, 6y44

PDB reference: complex with PLN-pS16, 6y40

## Figures and Tables

**Table 1:** Sigma grid screening containing 24 different crystallization conditions Each condition consists of (i) a constant concentration of the buffering agent HEPES-Na and CaCl_2_ and (ii) variation of the precipitant (PEG 400) and pH. Conditions were stored at 277 K.

Well	1	2	3	4	5	6	7	8	9	10	11	12
pH	7.1	7.1	7.1	7.1	7.1	7.1	7.3	7.3	7.3	7.3	7.3	7.3
PEG 400 (%)	24	25	26	27	28	29	24	25	26	27	28	29
CaCl_2_ (*M*)	0.19	0.19	0.19	0.19	0.19	0.19	0.19	0.19	0.19	0.19	0.19	0.19
HEPES-Na (m*M*)	95	95	95	95	95	95	95	95	95	95	95	95
pH	7.5	7.5	7.5	7.5	7.5	7.5	7.7	7.7	7.7	7.7	7.7	7.7
PEG 400 (%)	24	25	26	27	28	29	24	25	26	27	28	29
CaCl_2_ (*M*)	0.19	0.19	0.19	0.19	0.19	0.19	0.19	0.19	0.19	0.19	0.19	0.19
HEPES-Na (m*M*)	95	95	95	95	95	95	95	95	95	95	95	95
