# Supplementary material for: A new soaking procedure for X-ray crystallographic structural determination of protein–peptide complexes
Source: Acta Crystallogr F Struct Biol Commun. 2020 Sep 15;76(Pt 10):501–7. doi: 10.1107/S2053230X2001122X (PMC7531243; doi:10.1107/S2053230X2001122X)
Supplement: Supplementary file 1 [file f-76-00501-sup1.pdf]

STRUCTURAL BIOLOGY COMMUNICATIONS

Volume 76 (2020)

Supporting information for article:

A new soaking procedure for X-ray crystallographic structural determination of protein–peptide complexes

Alice Ballone, Roxanne A. Lau, Fabian P. A. Zweipfenning and Christian Ottmann

Table S1 Peptides used for soaking of the 14-3-3σ/c-Jun crystals (pS/T = phosphorylated serine/threonine).

PDB (1) corresponds to the new crystal structures from this paper, PDB (2) corresponds to crystal structures previously deposited in the PDB with the exception of cJun and PLN structures which were not based on any previous structure.

**Peptide**	**Organism**	**UniProt identifier**	**Peptide sequence**	**PDB (1)**	**PDB (2)**
**cJun-pS227**	*Homo sapiens*	A0A0B4KEI6	221-RNRVAA{pS227}SKCRKRK-234	6Y3V	N/A
**CaMKK2-pS100**	*Homo sapiens*	Q96RR4	97-RKL{pS100}LQER-104	6Y3O	6EWW
**CaMKK2-pS511**	*Homo sapiens*	Q96RR4	508-RSL{pS511}APGN-515	6Y8A	6FEL
**Caspase-2-pS139**	*Homo sapiens*	P42575	136-YDL{pS139}LPFP-143	6Y8B	6GKF
**Caspase-2-pS164**	*Homo sapiens*	P42575	161-VEH{pS164}LDNK-168	6Y8D	6GKG
**Gab2-pS210**	*Homo sapiens*	Q9UQC2	205-NARSA{pS210}FSQG-214	6Y3S	5EWZ
**Gab2-pT391**	*Homo sapiens*	Q9UQC2	286-IPRRN{pT391}LPAMDNS-399	6Y3R	5EWZ
**H+-ATPase-pT955**	*Nicotiana plumbaginifolia*	Q40409	QSY{pT995}TV	6YM	3E6Y
**MLF1-pS32**	*Homo sapiens*	P58340	29-RSF{pS32}EPFG-36	6Y8E	3UAL
**SOS1-pS1161**	*Homo sapiens*	Q07889	1155-PRRRPE{pS1161}APAESS-1168	6Y44	6F08
**PLN-pS16**	*Homo sapiens*	P26678	6-YLTRSAIRRA{pS16}TIEMP-21	6Y40	N/A

Table S2 Data collection and processing

Values in parentheses are for the highest resolution shell.

	**cJun-pS227 (6Y3V)**	**H+-ATPase-pT955 (6Y3M)**	**CaMKK2-pS100 (6Y3O)**
Source	DIAMOND BEAMLINE I24	DIAMOND BEAMLINE I03	DIAMOND BEAMLINE I03
Wavelength (Å)	0.9686	0.9763	0.973
Temperature (K)	100	100	100
Detector	DECTRIS PILATUS3 6M	DECTRIS EIGER2 XE 16M	DECTRIS EIGER2 XE 16M
Space group	C 2 2 21	C 2 2 21	C 2 2 21
a, b, c (Å)	82.68, 112.05, 62.58	82.25, 112.45, 62.88	82.51, 112.50, 62.43
α, β, γ (°)	90.00, 90.00, 90.00	90.00, 90.00, 90.00	90.00, 90.00, 90.00
Resolution range (Å)	66.531-1.499	34.42-1.5	45.53-1.5
Total No. of reflections	551488 (22223)	372080 (265)	364551 (317)
No. of unique reflections	46803 (2252)	36770 (207)	36489 (241)
Completeness (%)	99.0 (96.4)	78.69 (9.0)	78.25 (10.3)
Multiplicity	11.9 (9.9)	10.1 (1.3)	10 (1.3)
$\langle I/\sigma(I) \rangle$	3.7 (0.9)	19.7 (2.4)	18.4 (1.1)
R_{meas}	0.987 (10.670)	0.115 (0.232)	0.084 (0.400)
$R_{\text{p.i.m.}}$	0.324 (4.297)		
$CC_{1/2}$	0.483 (0.289)	1.0 (0.9)	1.0 (0.8)
Wilson B-factor (Å^2)	11.1	14.1	17.9

Table S3 Data collection and processing

Values in parentheses are for the highest resolution shell.

	**Gab2-pT391 (6Y3R)**	**Gab2-pS210 (6Y3S)**	**PLN-pS16 (6Y40)**
Source	DIAMOND BEAMLINE I03	DIAMOND BEAMLINE I03	RIGAKU MICROMAX-003
Wavelength (Å)	0.973	0.973	1.541
Temperature (K)	100	100	100
Detector	DECTRIS EIGER2 XE 16M	DECTRIS EIGER2 XE 16M	DECTRIS PILATUS 200K
Space group	C 2 2 21	C 2 2 21	C 2 2 21
a, *b*, *c* (Å)	82.44, 111.90, 62.35	82.45, 112.14, 62.71	82.30, 112.39, 62.57
α, β, γ (°)	90.00, 90.00, 90.00	90.00, 90.00, 90.00	90.00, 90.00, 90.00
Resolution range (Å)	66.37-1.50	66.43-1.95	41.81-1.59
Total No. of reflections	368117 (286)	279442 (13113)	181890 (579)
No. of unique reflections	33458 (211)	21568 (1054)	31591 (380)
Completeness (%)	72.04 (9.2)	100 (100)	80.2 (13.4)
Multiplicity	11.0 (1.4)	12.9 (12.4)	5.8 (1.5)
⟨$I/\sigma(I)$⟩	41.2 (7.3)	4.1 (0.9)	14.3 (2.4)
R_{meas}	0.049 (0.142)	0.348 (0.829)	0.078 (0.249)
$R_{\text{p.i.m.}}$			0.031 (0.152)
$CC_{1/2}$	1.0 (0.9)	1.0	0.0996 (0.937)
Wilson *B*-factor (Å^2)	12.4	16.7	9.7

Table S4 Data collection and processing

Values in parentheses are for the highest resolution shell.

	**Caspase-2-pS164 (6Y8D)**	**MLF1-pS32 (6Y8E)**	**Gab2-pT391 1h (6ZVE)**
Source	DIAMOND BEAMLINE I03	DIAMOND BEAMLINE I03	RIGAKU MICROMAX-003
Wavelength (Å)	0.973	0.973	1.541
Temperature (K)	100	100	100
Detector	DECTRIS EIGER2 XE 16M	DECTRIS EIGER2 XE 16M	DECTRIS PILATUS 200K
Space group	C 2 2 21	C 2 2 21	C 2 2 21
a, b, c (Å)	82.58, 112.71, 62.66	82.17, 112.05, 62.62	82.90, 113.03, 62.82
α, β, γ (°)	90.00, 90.00, 90.00	90.00, 90.00, 90.00	90.00, 90.00, 90.00
Resolution range (Å)	29.41-1.51	29.28-1.42	33.33-2.50
Total No. of reflections	514076 (7827)	726538 (28947)	55799 (3859)
No. of unique reflections	45100 (1757)	54757 (2720)	10057 (723)
Completeness (%)	97.6 (78.5)	100 (99.6)	96.4 (96.9)
Multiplicity	11.4 (4.5)	13.3 (10.6)	5.5 (5.3)
$\langle I/\sigma(I)\rangle$	64.3 (9.4)	43.2 (5.0)	4.0 (3.0)
R_{meas}	0.023 (0.132)	0.032 (0.458)	0.332 (0.359)
$R_{\text{p.i.m.}}$	0.006 (0.060)	0.009 (0.139)	0.147 (0.155)
$CC_{1/2}$	1.0 (0.986)	1.000 (0.942)	0.891 (0.871)
Wilson B-factor (Å^2)	15.4	16.2	6.6

Table S5 Data collection and processing

Values in parentheses are for the highest resolution shell.

	**Gab2-pT391 24h (6ZVB)**	**Gab2-pT391 48h (6ZVC)**	**Gab2-pT391 96h (6ZVD)**
Source	RIGAKU MICROMAX-003	RIGAKU MICROMAX-003	RIGAKU MICROMAX-003
Wavelength (Å)	1.541	1.541	1.541
Temperature (K)	100	100	100
Detector	DECTRIS PILATUS 200K	DECTRIS PILATUS 200K	DECTRIS PILATUS 200K
Space group	C 2 2 21	C 2 2 21	C 2 2 21
a, *b*, *c* (Å)	82.40, 111.91, 62.47	82.57, 112.51, 62.66	82.35, 111.98, 62.43
α, β, γ (°)	90.00, 90.00, 90.00	90.00, 90.00, 90.00	90.00, 90.00, 90.00
Resolution range (Å)	33.18-2.50	28.35-2.51	41.17-2.50
Total No. of reflections	55778 (4343)	62686 (4722)	64144 (4865)
No. of unique reflections	9816 (730)	10297 (735)	10293 (745)
Completeness (%)	96.0 (98.6)	99.6 (98.5)	100.0 (100.0)
Multiplicity	5.7 (5.9)	6.1 (6.4)	6.2 (6.5)
$\langle I/\sigma(I)\rangle$	12.8 (9.7)	19.0 (14.5)	26.9 (18.5)
R_{meas}	0.093 (0.116)	0.074 (0.098)	0.054 (0.087)
$R_{\text{p.i.m.}}$	0.042 (0.049)	0.029 (0.054)	0.021 (0.034)
$CC_{1/2}$	0.990 (0.989)	0.997 (0.996)	0.999 (0.996)
Wilson *B*-factor (Å^2)	7.6	7.9	11.2

Table S6 Data collection and processing

Values in parentheses are for the highest resolution shell.

	**SOS1-pS1161 (6Y44)**	**CaMKK2-pS511 (6Y8A)**	**Caspase-2-pS139 (6Y8B)**
Source	RIGAKU MICROMAX-003	DIAMOND BEAMLINE I03	DIAMOND BEAMLINE I03
Wavelength (Å)	1.541	0.973	0.973
Temperature (K)	100	277	277
Detector	DECTRIS PILATUS 200K	DECTRIS EIGER2 XE 16M	DECTRIS PILATUS 200K
Space group	C 2 2 21	C 2 2 21	C 2 2 21
a, *b*, *c* (Å)	81.77, 111.63, 62.79	82.24, 112.14, 62.82	82.80, 112.96, 62.45
α, β, γ (°)	90.00, 90.00, 90.00	90.00, 90.00, 90.00	90.00, 90.00, 90.00
Resolution range (Å)	29.81-1.71	29.325-1.5	30.05-1.54
Total No. of reflections	422523 (21039)	505896 (7060)	434737 (3960)
No. of unique reflections	31324 (1623)	45770 (1763)	42300 (1084)
Completeness (%)	99.9 (98.0)	97.9 (78.0)	97.6 (51.4)
Multiplicity	13.5 (13)	11.1 (4.0)	10.3 (3.7)
$\langle I/\sigma(I)\rangle$	28.2 (3.3)	26.5 (3.6)	14.3 (2.2)
R_{meas}	0.053 (0.814)	0.061 (0.420)	0.158 (0.631)
$R_{\text{p.i.m.}}$	0.014 (0.222)	0.017 (0.200)	0.046 (0.296)
$CC_{1/2}$	1.0 (0.923)	0.999 (0.870)	0.996 (0.660)
Wilson *B*-factor (Å^2)	25.5	15.9	15.9

Table S7 Structure refinement

Values in parentheses are for the highest resolution shell.

	**cJun-pS227 (6Y3V)**	**H+-ATPase-pT955 (6Y3M)**	**CaMKK2-pS100 (6Y3O)**
Resolution range (Å)	66.531-1.499	34.42-1.5	45.53-1.5
Final R_{work}	0.193	0.163	0.173
Final R_{free}	0.221	0.197	0.198
No. of non-H atoms			
Protein	1845	1850	1840
Ligand	10	37	44
Water	462	479	388
R.m.s. deviations			
Bonds (Å)	0.32	0.33	0.33
Angles (°)	0.50	0.49	0.49
Average B, all atoms (Å^2)	17.0	18.0	21.0
Ramachandran plot			
Favored regions (%)	98	99	99
Allowed (%)	2	1	1
Disallowed (%)	0	0	0

Table S8 Structure refinement

Values in parentheses are for the highest resolution shell.

	**Gab2-pT391 (6Y3R)**	**Gab2-pS210 (6Y3S)**	**PLN-pS16 (6Y40)**
Resolution range (Å)	66.37-1.50	66.43-1.95	41.81-1.59
Final R_{work}	0.182	0.173	0.168
Final R_{free}	0.22	0.211	0.207
No. of non-H atoms			
Protein	1855	1826	1920
Ligand	84	43	46
Water	433	432	517
R.m.s. deviations			
Bonds (Å)	0.33	0.35	0.36
Angles (°)	0.52	0.48	0.49
Average B, all atoms (Å^2)	18	20	12
Ramachandran plot			
Favored regions (%)	98	98	99
Allowed (%)	2	2	1
Disallowed (%)	0	0	0

Table S9 Structure refinement

Values in parentheses are for the highest resolution shell.

	**Caspase-2-pS164 (6Y8D)**	**MLF1-pS32 (6Y8E)**	**Gab2-pT391 1h (6ZVE)**
Resolution range (Å)	29.41-1.51	29.28-1.42	33.42-2.51
Final R_{work}	0.175	0.174	0.247
Final R_{free}	0.197	0.201	0.265
No. of non-H atoms			
Protein	1899	1858	1804
Ligand	37	63	84
Water	351	367	143
R.m.s. deviations			
Bonds (Å)	0.33	0.33	0.72
Angles (°)	0.51	0.51	0.64
Average *B*, all atoms (Å^2)	21.0	22.0	8.0
Ramachandran plot			
Favored regions (%)	98	99	98
Allowed (%)	2	1	2
Disallowed (%)	0	0	0

Table S10 Structure refinement

Values in parentheses are for the highest resolution shell.

	**Gab2-pT391 24h (6ZVB)**	**Gab2-pT391 48h (6ZVC)**	**Gab2-pT391 96h (6ZVD)**
Resolution range (Å)	33.18-2.51	28.35-2.50	41.17-2.50
Final R_{work}	0.200	0.171	0.174
Final R_{free}	0.263	0.229	0.234s
No. of non-H atoms			
Protein	1852	1845	1820
Ligand	84	84	84
Water	174	195	180
R.m.s. deviations			
Bonds (Å)	0.73	0.73	0.72
Angles (°)	0.76	0.62	0.63
Average B, all atoms (Å^2)	11.0	11.0	14.0
Ramachandran plot			
Favored regions (%)	98	98	98
Allowed (%)	2	2	2
Disallowed (%)	0	0	0

Table S11 Structure refinement

Values in parentheses are for the highest resolution shell.

	**SOS1-pS1161 (6Y44)**	**CaMKK2-pS511 (6Y8A)**	**Caspase-2-pS139 (6Y8B)**
Resolution range (Å)	29.81-1.71	29.325-1.5	30.05-1.54
Final R_{work}	0.174	0.171	0.18
Final R_{free}	0.202	0.201	0.212
No. of non-H atoms			
Protein	1916	1916	1847
Ligand	69	51	71
Water	238	394	363
R.m.s. deviations			
Bonds (Å)	0.36	0.32	0.32
Angles (°)	0.48	0.50	0.50
Average *B*, all atoms (Å^2)	30.0	21.0	20.0
Ramachandran plot			
Favored regions (%)	98	98	98
Allowed (%)	2	2	2
Disallowed (%)	0	0	0

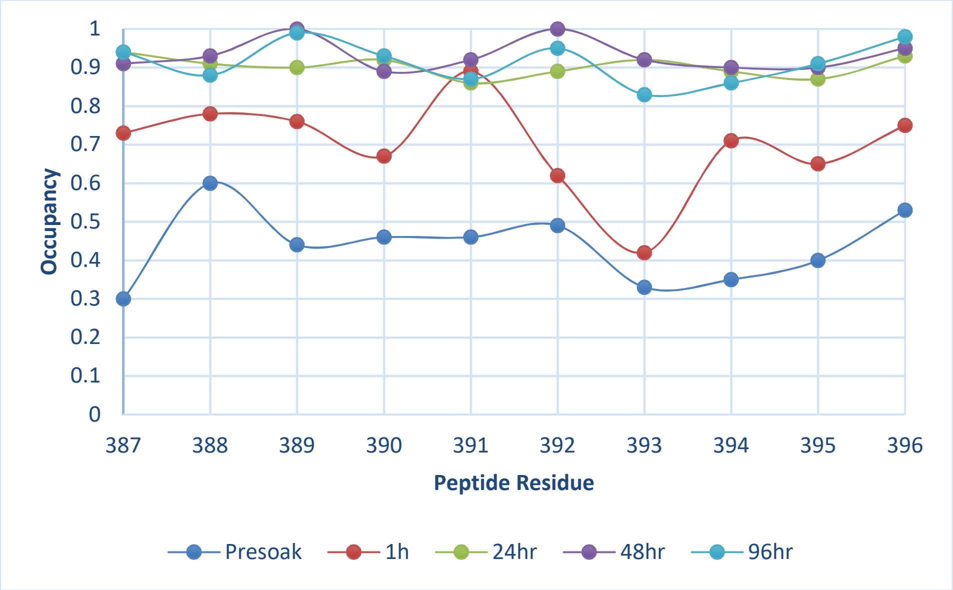

Figure S1 Occupancy Plot. Full occupancy of the peptide is achieved at 48h for all the amino acids surrounding pT391 of Gab2 peptide.

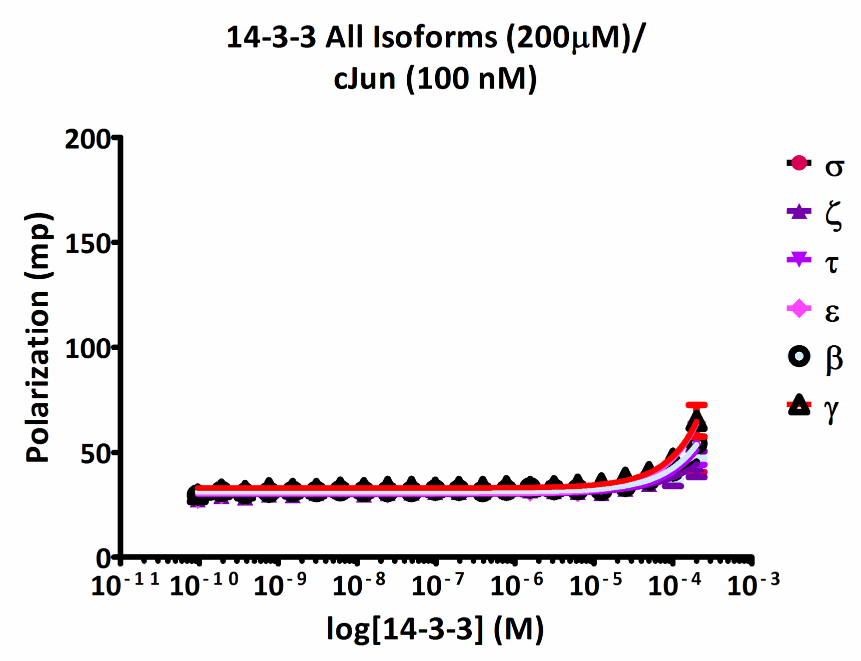

Figure S2 Binding between cJun peptide to all human 14-3-3 isoforms in a fluorescence polarization assay. Background polarization was subtracted from all values. Mean of three experiments; SD error bars are smaller than the data point symbols. Fluorescence polarization was chosen as the assay format to consider 14-3-3 isoform dependence. The 13-mer cJun peptide was labeled on the N-terminus with Fluorescein isothiocyanate (FITC) and used as a tracer to measure phospho-site binding affinity. The result from the assays showed the motif to be a weak binder to all

seven isoforms. The assay is in line with the results obtained by means of X-ray protein crystallography. The protocol followed to carry out these data was previously published in (Ballone, Centorrino, Wolter *et al.*, 2018*c*).
